# Should Panelists Refrain from Wearing a Personal Fragrance Prior to Sensory Evaluation? The Effect of Using Perfume on Olfactory Performance

**DOI:** 10.3390/foods11030428

**Published:** 2022-02-01

**Authors:** Thadeus L. Beekman, Kaushik Luthra, Shady Afrin Jeesan, Rebecca Bowie, Han-Seok Seo

**Affiliations:** Department of Food Science, University of Arkansas, 2650 North Young Avenue, Fayetteville, AR 72704, USA; tlbeekma@uark.edu (T.L.B.); kluthra@uark.edu (K.L.); sjeesan@uark.edu (S.A.J.); rlbowie@uark.edu (R.B.)

**Keywords:** olfactory, threshold, discrimination, identification, Sniffin’ Sticks, fragrance, perfume, sensory

## Abstract

It is typically recommended that panelists should refrain from wearing personal fragrances, such as perfume or cologne, prior to sensory evaluation. Interestingly, no study has been reported as to whether panelists’ perceptions of test samples could be affected by personal fragrances worn by themselves. The objective of this study was, therefore, to determine the effect of such a personal fragrance on olfactory performance. Nineteen untrained participants were screened, recruited for, and underwent the Sniffin’ Sticks test designed for measuring olfactory performances that included the odor threshold, discrimination, and identification (TDI). The olfactory performance tasks were conducted under three fragrance level conditions: (1) control (no fragrance), (2) just-about-right (JAR), and (3) excessive, with a preliminary study used to identify both the JAR and excessive fragrance levels. The results showed that the odor discrimination, odor threshold, and combined TDI performances were significantly lowered in the two conditions with the perfume fragrance, while the odor identification performance exhibited no significant differences across all three conditions. These findings provide empirical evidence that even low to moderate levels of personal fragrance can significantly reduce individuals’ olfactory capabilities, possibly subsequently altering the perception of test samples during sensory evaluation.

## 1. Introduction

There is a commonly held belief that participants should not wear any sort of fragrance prior to the sensory evaluation of test samples. Although this condition, to have participants refrain from wearing personal fragrances such as perfume or cologne, is specifically mentioned in foundational texts [1,2]; there is a dearth of research offering solid support to the idea that personal fragrance should be avoided within sensory testing. Even greater scarcity is found of prior work that details any effect of the level of personal fragrance on the sensory perception of test samples. Considering the idiosyncratic preferences of consumers with respect to their preferred fragrance levels, there may be genetic influences on fragrance level preferences [3,4,5]. Although there has been earlier research suggesting that cigarette smoking and its residual odors could have an impact on the sensory perception of food samples [6], when we consider that more than half of adults report regularly wearing some sort of personal fragrance [7], it seems highly necessary to develop a solid understanding of how applied personal fragrances can impact sensory perception. Allen et al. [5] have revealed that participants better performed a triangle test of body odor samples under a no-fragrance condition compared to under fragrance conditions (i.e., wearing either their own fragrance or an assigned fragrance). It should also be noted that perfume and cologne are not the only sources of personal fragrance; deodorant, shampoo, hairspray, and even laundry detergent, could be sources of fragrance found on consumers, meaning that half the population could be a highly conservative estimate of all consumers exhibiting such a quality.

As the sense of smell plays a key role in flavor perception and food acceptance [8,9,10], this study has focused on the effect on olfactory performance of wearing personal fragrance. One of the physiological factors related to a potential effect of personal fragrance on olfactory performance would be adaptation, defined as a “decrease in sensitivity or response to an odor stimulus following repetitive stimulation” [11], because the odors of personal fragrances sprayed on the skin or clothing do not usually go away quickly [2]. Due to this adaptation effect, the olfactory abilities and the subsequent sensory-evaluation performances of consumer panelists would be predictably skewed when wearing personal fragrances.

The Sniffin’ Sticks battery is a well-validated measurement tool for addressing olfactory capabilities with respect to the odor threshold, odor discrimination, and odor identification [12,13]. These three tasks, distinguishable as individual constructs, generally require independent mental processes relative to one another [14,15]. Since such separated cognitive constructs are also observed in olfactory performance [16,17], the investigation of how personal fragrance impacts the olfactory threshold, discrimination, and identification capabilities individually, as well as together through the combined TDI score, offers an even greater degree of understanding to the field.

An important caveat to consider in this area of research is the presence of individual differences with respect to odor sensitivity. Previous research has revealed numerous factors contributing to variation in odor sensitivity, including demographic profiles (e.g., gender and age) [13,18], hunger/satiety [19], personality traits [20], and other environmental variables [21]. For example, as people age, they become less sensitive to odors, such as personal fragrances [13], which may lead older individuals to apply higher levels of personal fragrance to achieve the same intensity of fragrance to which they were previously accustomed earlier in life. For younger individuals, or others with greater olfactory sensitivities, an application of a greater amount of personal fragrance could be perceived as an excessively or even unpleasantly strong scent, potentially resulting in discomfort or possible sensory and pulmonary irritation in more sensitive individuals [22]. More research exists on age-related differences, providing further details of how aging is accompanied by changes in body odor and scent perception, possibly contributing to older individuals choosing to increase the amount of personal fragrance applied [23,24]. Another area to be considered in olfactory capabilities is gender differences. Earlier studies have detailed how females have consistently displayed greater olfactory capabilities than male counterparts; although a recent meta-analysis performed through a comprehensive consideration of the associated literature, found that the effect sizes and olfactory performance differences between males and females may be relatively small [25].

In view of all these factors, the over-arching goal of this study was to identify the impact of personal fragrance on individuals’ olfactory performances, especially the threshold, discrimination, and identification, as functions of fragrance presence (i.e., presence vs. absence) and the fragrance intensity level (i.e., absence vs. just-about-right vs. excessive). These findings provide current and future researchers with specific evidence and support as to why participants should be prevented from wearing a fragrance in sensory testing.

## 2. Materials and Methods

The protocol (No. 1808138603) used in this study was approved by the Institutional Review Board (IRB) of the University of Arkansas (Fayetteville, AR, USA). Prior to participation, the experimental procedure was explained to all participants, and an informed written consent indicating voluntary participation was obtained from each participant.

### 2.1. Preliminary Testing

The main study considered three perfume fragrance conditions: no fragrance (control), just-about-right (JAR) intensity, and excessive intensity of fragrance. To determine the amounts of the perfume needed to achieve both the JAR and the excessive levels of fragrance, preliminary testing was conducted prior to the main study. During the preliminary testing, it was determined that one spray of perfume fragrance was equivalent to a mass of 0.054 g (standard deviation (SD) = 0.003 g). It was also determined that fragrance levels of 3 (approximately, 0.162 g) and 10 (approximately, 0.540 g) sprays were perceived as the JAR and excessive levels of fragrance, respectively.

### 2.2. Participants

Participants were initially screened and recruited from the Northwest Arkansas community through the University of Arkansas Sensory Science Center (Fayetteville, AR, USA) consumer profile database. Participants with known food allergies or clinical histories of major diseases (e.g., diabetes or cancer) were not included in this study. Participants were also screened for self-reported olfactory abilities, with any individuals reporting any difficulties or diminished smelling capabilities being excluded from the study. A total of 20 participants were recruited, with all but one completing the entire study. Due to the incomplete dataset, the drop-outs data were not included in the analysis or its results, leaving a total of 19 participants (10 females and 9 males) with a mean age of 33 years (SD = 9 years). A power analysis was conducted that supports a sample size of 19 within this study, while maintaining a minimum power level of 0.80. All participants were asked to refrain from eating, drinking, or cigarette smoking for two hours prior to each test, and were also instructed not to wear any fragrances of their own to the test sessions. Data were collected from October to November 2019 (i.e., prior to the COVID-19 pandemic).

### 2.3. Perfume Sample and Fragrance Preparation

In all three sessions: no fragrance, JAR fragrance, and excessive fragrance, participants were asked to wear a black, wait-staff style apron (Cheflux Choice, Hebron, KY, USA) for the duration of the study. The perfume was applied at the JAR (3 sprays) and excessive levels (10 sprays) on the chest areas of the aprons in a separate room directly before each session. To reduce the potential variation between the researchers, with respect to the possible mixture of the perfume fragrance and body odors [26,27], the perfume was applied to their aprons, and not to the researchers’ bodies [28]. After applying the perfume to the apron, the researcher immediately provided the participant with the apron. The perfume used throughout the study was Marc Jacobs Decadence (Marc Jacobs Decadence Eau De Parfum Spray, Marc Jacobs, New York City, NY, USA), chosen because prior research within our lab had determined that both males and females perceive this fragrance as gender-neutral [28]; previous research has also revealed that feminine and masculine fragrances can differently influence participants’ sensory perception [29,30].

### 2.4. Procedure

Each participant completed three separate test sessions, one week apart. The sessions were conducted in a balanced and randomized order across the participants. Multiple testing-rooms were used to provide one-on-one sessions between the researcher and a participant. All the rooms used for testing were in air-conditioned and ventilated environments, with additional time of one hour allotted after each session to allow for further ventilation. Once a participant had been seated for her/his test session, the researcher provided an overview of the session procedure and how to perform the desired olfactory tasks using the Sniffin’ Sticks battery (Burghardt, Wedel, Germany). The researcher then prepared the corresponding apron–fragrance treatment combination in a separate room, and then instructed the participant to wear it for the duration of the session. The researcher then initiated the Sniffin’ Sticks testing in the order of the threshold, discrimination, and identification tasks, with the participant being blindfolded during the odor threshold and discrimination tasks [12]. The threshold, discrimination, and identification scores were calculated separately following the prior guidelines, then combined to produce a total TDI score [12].

### 2.5. Statistical Analysis

Data were analyzed using JMP Pro 16 software (SAS Institute Inc., Cary, NC, USA). A two-way analysis of variance (ANOVA), treating “fragrance condition” and “participant” as fixed and random effects, respectively, was conducted to determine whether olfactory performances, such as the odor threshold, odor discrimination, and odor identification, might differ under the three fragrance conditions. If a significant effect was identified, post hoc multiple pairwise comparisons between fragrance conditions were conducted using Tukey’s Honestly Significant Difference (HSD) tests. A statistical significance was defined to exist when *p* < 0.05.

## 3. Results

**Figure 1:**
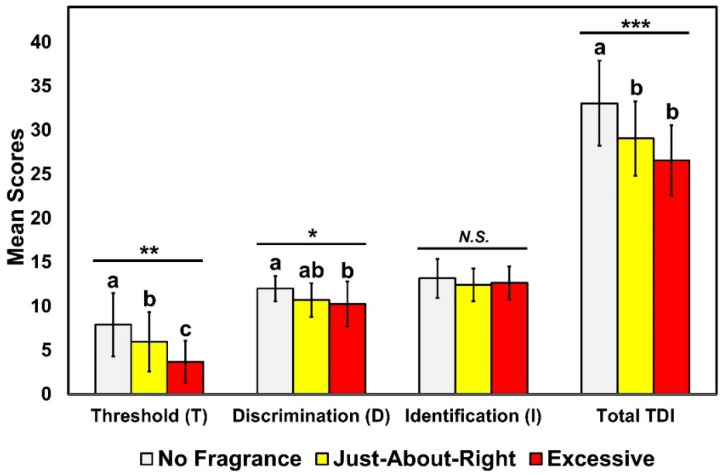
Mean score comparisons among the three fragrance conditions: no fragrance, just about right (JAR) fragrance, and excessive fragrance, with respect to the odor threshold (T), odor discrimination (D), and odor identification (I) tasks, along with a combined TDI score, for the Sniffin’ Sticks test. Error bars represent standard deviations. *, **, and *** represent a significant difference at *p* < 0.05, *p* < 0.01, and *p* < 0.001, respectively. *N.S.* represents no significant difference at *p* < 0.05. The different letters above the bars within each olfactory task category indicate a significant difference at *p* < 0.05.

Figure 1 shows the mean comparisons among the three fragrance conditions with respect to the odor threshold (T), odor discrimination (D), and odor identification (I), along with their combined TDI score. There were significant effects of the fragrance condition both in the threshold scores (*F* = 14.23, *p* < 0.001) and the discrimination scores (*F* = 4.89, *p* = 0.01). More specifically, for the odor threshold task, the mean (±SD) score under the no fragrance condition (7.89 ± 3.58) was significantly greater than the mean score under the JAR fragrance condition (5.95 ± 3.37), and both were significantly greater than the mean score under the excessive fragrance condition (3.67 ± 2.38). For the odor discrimination task, the mean (±SD) score under the no fragrance condition (12.00 ± 1.45) was significantly greater than the mean score for the excessive fragrance condition (10.26 ± 2.54), while the mean score for the JAR fragrance condition (10.68 ± 1.92) was not significantly different from that under either the no fragrance or the excessive fragrance condition. However, for the odor identification task, there were no significant differences in the mean scores among the three fragrance conditions (*F* = 2.28, *p* = 0.12): no fragrance (13.16 ± 2.19), JAR (12.42 ± 1.84), and excessive fragrance (12.63 ± 1.89).

The mean comparisons of the combined TDI scores revealed similar trends among the three fragrance conditions (*F* = 20.15, *p* < 0.001). As shown in Figure 1, the no fragrance condition exhibited a significantly higher mean score (33.05 ± 4.83) than both the JAR fragrance (29.05 ± 4.23) and the excessive fragrance (26.57 ± 4.01) conditions, that were not significantly different from one another.

## 4. Discussion

The results of this study suggest that wearing personal fragrance significantly lowers individuals’ olfactory capabilities, specifically in the odor threshold and discrimination tasks, with no performance differences between fragrance conditions within the identification task (Figure 1). One caveat to keep in mind is that a standardized personal fragrance was used across all participants, meaning that they did not choose their own personal fragrance. The degree of applied fragrance also had a greater impact on the threshold than on the discrimination performance of participants. Combining the three olfactory tasks into an overall measure of olfactory performance, a TDI score produced similar results with both the JAR and excessive fragrance conditions, inducing a significantly lower score compared to the no fragrance condition. These combined findings resulted in the capability for fully addressing the main research question, providing validating evidence for the common practice of requiring participants to refrain from wearing personal fragrances during sensory evaluations.

Olfactory adaptation resulting from constant exposure to perfume fragrance is one of the two plausible reasons contributing to the diminished threshold and discrimination performances under both fragrance conditions. The other reason, related to a lowered olfactory performance under the fragrance conditions, would be a masking effect because of the personal fragrance, notably in the excessive condition, possibly masking the lower-intensity scents from the Sniffin’ Sticks test [31,32]. It should be noted that in the methodology used by the Sniffin’ Sticks TDI set, the odor threshold task was conducted first, suggesting that the findings here might be related to the possible diminution of the olfactory capabilities minutes after being exposed to the scent. Other research has mirrored this finding, supporting the notion that even relatively short, constant exposure to a scent can impact consumer olfactory capabilities [33].

Comparatively, it is of interest to explore why the Sniffin’ Sticks identification task did not reflect lowered scores when participants were wearing personal fragrances. One reason for the lack of a personal fragrance effect on the identification task performance could be the intensity of the identification tasks’ Sniffin’ Sticks. Compared to the odor sticks used for the threshold or discrimination tasks, the odor sticks used for the odor identification tasks exhibited stronger odor intensities and a greater number of unique odors, thereby lessening the effect of the perfume fragrance on the odor identification performance. Olfactory adaptation research has indicated that such adaptation may only influence scents of lower intensity, meaning that the more highly intense scents of the odor identification sticks had no issues with respect to being detected by participants in the fragrance treatments [32,33]. Other studies also indicate that identification and discrimination tasks involve two different cognitive processes, thus offering additional insight as to why performance differences were not seen in the identification task between the fragrance conditions [14,15,16,17].

The olfactory system is universally known to interact very closely with flavor perception, and is also closely associated with memories, emotions, and other senses that consumers may use in forming their overall opinions of products and experiences [34,35]. Because of the high degree of importance of the olfactory system, when consumers are interacting with or evaluating test samples, their perceptions, acceptances, and opinions should not be influenced by their personal fragrance regardless of the test samples. Such findings not only apply to consumer panelists in sensory evaluation, but also, with arguably equal or greater importance, to trained descriptive panelists. Specifically, a properly functioning sense of olfaction is critical for optimal descriptive panel performance, and the presence of even one person with lowered olfactory capabilities can skew descriptive panel results [2]. Due to such training potentially impacting how trained panelists evaluate scent stimuli, the effect of personal fragrance on trained panelists’ olfactory abilities may not be fully equivalent, yet a diminished olfactory ability is still predicted and would be interesting to consider in future research. This study’s findings also provide insight related to environmental odor stimuli influencing consumer perception [28,36,37]. Although this study was conducted via one-on-one assessments, in more ecologically valid scenarios, one consumer’s personal fragrance may introduce an unintentional, and most likely undesired, environmental scent. To better address the variables of the personal preference for the type and level of fragrance, similar studies could also directly build on this one by instructing participants to apply their own fragrance [5,27]. It is also known that various levels of ambient scents, as opposed to applied personal fragrances, can separately impact consumer olfactory perception and performance [38,39,40,41]. One potential limitation of this study is that there was a standard fragrance used across all participants. This aspect could induce the effects seen here on olfactory performance to be less specific to personal fragrance, as participants may not have perceived the applied fragrance in this study to be more of an environmental scent. Due to this possible limiting factor, the investigation of how participants apply their own personal fragrance could offer clarifying insight. Additional research topics suggested by this study are to consider the impact of personal fragrance on retro-nasal olfactory performance [42], or how consumer emotions are involved in the effect of personal fragrance on sensory evaluation performances [35]. These additional points help to highlight the importance of considering the impact of personal fragrance on olfactory abilities.

## 5. Conclusions

Connecting diverse areas of olfaction research with the results of this study offers a straightforward conclusion: personal fragrance, even at low to moderate levels, significantly lowers individuals’ olfactory capabilities, especially the odor threshold and odor discrimination. Collectively, these results concurrently represent a significant impact for industry applications and areas of future study. As this study provided initial evidence detailing how personal fragrance sprayed on consumers can negatively impact their olfactory capabilities, areas of sensory, marketing, and consumer research (both food and non-food) can directly utilize these findings. It is crucial for future research to understand how this effect occurs within more realistic consumer-sample situations, while also considering additional variables that may interact with personal fragrance and influence not only olfactory capabilities, but also the overall perceptual capabilities of consumers when interacting with test samples. Specifically, additional studies can offer further insight into how other individuals within the environment are impacted by an individual wearing personal fragrance, how these findings apply to settings outside of a sensory lab when participants can apply their own fragrance, or the impact of personal fragrance on retro nasal olfaction. Researchers should also consider that aprons were worn in the current study and that changing the application area or clothing item (e.g., apron vs. lab coat) worn by participants could impact how they perceive the applied fragrance and, subsequently, how their olfactory abilities are impacted by the fragrance. Separately examining each of these topics would further add to the understanding of how personal fragrance can impact human olfactory perception and performance.

## Data Availability

The data are not publicly available due to the Institutional Review Board protocol guideline.
